# Correlation of BACH1 and Hemoglobin E/Beta-Thalassemia Globin Expression

**DOI:** 10.4274/tjh.2014.0197

**Published:** 2016-02-17

**Authors:** Tze Yan Lee, Logeswaran Muniandy, Lai Kuan Teh, Maha Abdullah, Elizabeth George, Jameela Sathar, Mei I Lai

**Affiliations:** 1 Universiti Putra Malaysia Faculty of Medicine and Health Sciences, Department of Pathology, Serdang, Malaysia; 2 Universiti Putra Malaysia Faculty of Medicine and Health Sciences, Genetic and Regenerative Medicine Research Centre, Serdang, Malaysia; 3 Ampang Hospital, Clinic of Hematology, Selangor, Malaysia

**Keywords:** BACH1, Gene expression, Hemoglobin E/β-thalassemia, oxidative stress, Red blood cell parameters

## Abstract

**Objective:**

The diverse clinical phenotype of hemoglobin E (HbE)/β-thalassemia has not only confounded clinicians in matters of patient management but has also led scientists to investigate the complex mechanisms involved in maintaining the delicate red cell environment where, even with apparent similarities of α- and β-globin genotypes, the phenotype tells a different story. The BTB and CNC homology 1 (BACH1) protein is known to regulate α- and β-globin gene transcriptions during the terminal differentiation of erythroid cells. With the mutations involved in HbE/β-thalassemia disorder, we studied the role of BACH1 in compensating for the globin chain imbalance, albeit for fine-tuning purposes.

**Materials and Methods:**

A total of 47 HbE/β-thalassemia samples were analyzed using real-time quantitative polymerase chain reaction and correlated with age, sex, red blood cell parameters, globin gene expressions, and some clinical data.

**Results:**

The BACH1 expression among the β-thalassemia intermedia patients varied by up to 2-log differences and was positively correlated to age; α-, β-, and γ-globin gene expression level; and heme oxygenase 1 protein. BACH1 was also negatively correlated to reticulocyte level and had a significant correlation with splenectomy.

**Conclusion:**

This study indicates that the expression of BACH1 could be elevated as a compensatory mechanism to decrease the globin chain imbalance as well as to reduce the oxidative stress found in HbE/β-thalassemia.

## Abstract (tr)

**Amaç:**

Hemoglobin E (HbE)/β-talaseminin çeşitli klinik fenotipleri klinisyenlerin hasta yönetimi esnasında zihinlerini karıştırmakla kalmamış, α- ve β-globin genotiplerinde bariz benzerlikler varken fenotiplerde farklılıklar bulunduğundan bilim insanlarının hassas eritrosit çevrenin muhafaza edilmesinde yer alan karmaşık mekanizmaları incelemelerine de ön ayak olmuştur. BTB ve CNC homoloji 1 (BACH1) proteininin eritroid hücrelerin son farklılaşması sırasında α- and β-globin gen transkripsiyonlarını ayarladığı bilinmektedir. HbE/β-talasemi hastalığındaki mutasyonlar ile her ne kadar ince ayar amaçlı ise de BACH1’in globin zincir dengesizliğini kompanse etmedeki rolünü inceledik.

**Gereç ve Yöntemler:**

Toplam 47 HbE/β-talasemi örneği gerçek zamanlı kantitatif polimeraz zincir reaksiyonu ile incelendi ve yaş, cinsiyet, eritrosit değişkenleri, globin gen sunumları ve bazı klinik veriler ile korele edildi.

**Bulgular:**

β-talasemi intermedia hastalarındaki BACH1 sunumu 2-log’a kadar farklılık göstermekteydi ve yaş; α-, β- ve γ-globin gen sunum düzeyleri; ve hem oksijenaz 1 proteini ile pozitif korelasyonu vardı. Ayrıca BACH1’in retikülosit düzeyi ile negatif korelasyonu vardı ve splenektomi ile anlamlı korelasyonu bulunmaktaydı.

**Sonuç:**

Bu çalışma hem HbE/β-talasemide bulunan oksidatif stresi hem de globin zincir dengesizliğini azaltmak için BACH1 sunumunun kompansasyon mekanizması olarak artabileceğini göstermiştir.

## INTRODUCTION

Hemoglobin E (HbE) is a highly common hemoglobin variant in Asia. The substitution of G>A at codon 26 of the β-globin gene not only causes a reduced and structurally abnormal HbE production but also activates a cryptic splice site, leading to abnormal messenger ribonucleic acid (mRNA) processing. The heterozygous state of HbE is asymptomatic with minimal morphological red cell changes, and homozygous HbE at most displays mild anemia. However, when this variant is co-inherited with β-thalassemia, the clinical spectrum ranges from mild anemia to severe thalassemia intermedia [1,2].

This complex phenotype is affected not only by free α-globin chain precipitations causing membrane damage and increased reactive oxygen species, but also by various secondary and tertiary modifiers including variable compensatory mechanisms available in the red cell environment. This is evident particularly in families with seemingly similar α/β genotypes but discordant phenotypes [3].

BACH1 is a heme-binding factor that regulates multiple gene expressions. By forming a multivalent deoxyribonucleic acid (DNA)-binding complex in the enhancer regions of targeted genes, BACH1 represses transcription of its target genes. However, this function is inhibited in the presence of heme [4,5]. BACH1 plays an important role in the expression of heme-responsive genes like α- and β-globin, which are the key proteins in HbE/β-thalassemia, as well as in the regulation of heme oxygenase 1 (HO-1), a stress-responsive protein [5,6,7].

As BACH1 is able to suppress the expression of α- and β-globin genes under physiological conditions, the expression of BACH1 in HbE/β-thalassemia is of interest. A study by De Franceschi et al. showed a reduced intracellular heme content in late β-thalassemic precursors, which leads to an increased expression of the BACH1 gene [8]. Perhaps the increased BACH1 expression may play a compensatory role to reduce α/β-globin chain imbalance, thereby decreasing the oxidative stress produced by the imbalance. Thus, this study investigates the correlation of BACH1 to HbE/β-thalassemia parameters, where any effects of BACH1 would most likely be manifested.

## MATERIALS AND METHODS

### Study Population

HbE/β-thalassemia patients from the Ampang Hospital Thalassemia Clinic were recruited for this study. The HbE/β-thalassemia patients in our cohort were either transfusion-independent or previously had transfusion record of not less than 3 months prior to recruitment for this study. Ethical approval was obtained from the Medical Research and Ethics Committee, Ministry of Health Malaysia (NMRR-10-1177-6947), and the Medical Research Ethics Committee, Faculty of Medicine and Health Sciences, Universiti Putra Malaysia (UPM/FPSK/PADS/T7-MJKEtikaPer/F01). All subjects had given their informed consents prior to blood collection and anonymity of all data was made possible by numerical identification throughout the study.

### Laboratory Studies

#### Full Blood Count, High-Performance Liquid Chromatography, and Plasma Ferritin Study

Collection of blood samples were done in BD Vacutainer spray-dried K2EDTA tubes and BD Vacutainer freeze-dried lithium heparin tubes (Becton, Dickinson and Company, Franklin Lakes, NJ, USA) and full blood count indices were analyzed using a Sysmex 5000i Automated Hematology Analyzer (Sysmex, Kobe, Japan) according to the manufacturer’s protocol. The screening of HbE/β-thalassemia was performed using the VARIANT II β-Thalassemia Short Program on the VARIANT II Hemoglobin Testing System (Bio-Rad, Hercules, CA, USA). Plasma ferritin analysis was performed using the Tina-Quant ferritin assay (Cobas, Roche Diagnostics GmbH, Mannheim, Germany) on the Hitachi 902 Automatic Chemistry Analyzer (Hitachi, Ibaraki, Japan) according to the manufacturer’s protocol.

### Genomic Studies

Genomic DNA was extracted using the QIAamp Blood Midi Kit (QIAGEN GmbH, Hilden, Germany). Amplification refractory mutation system polymerase chain reaction (PCR) using primer sequences and PCR protocol modified from Old was performed to identify and confirm HbE and β-thalassemia mutations for each sample [9]. The β-thalassemia mutations characterized were codon 19 (A>G), IVS I-5 (G>C), IVS I-1 (G>T), codon 41/42 (-TCTT), and IVS II-654 (C>T). Genomic sequencing was done on uncharacterized samples. Co-inheritance of α-thalassemia was determined by --SEA,/-α3.7, and /-α4.2 mutation screening to minimize the occurrence of confounding factors for this study. Xmn1 polymorphisms were determined as previously described by Wong et al. [10]. Primers covering the exons of the BACH1 gene were used to sequence the gene. Primers and PCR conditions for the BACH1 gene are available upon request.

### Expression Analysis

Ribonucleic acid (RNA) extraction from peripheral reticulocytes was performed as described previously by Lai et al. [11]. Expression levels of BACH1, HO-1, and α-, β-, and γ-globin genes were quantified using real-time quantitative reverse transcription-PCR (Applied Biosystems, Warrington, UK). Glyceraldehyde 3-phosphate dehydrogenase (GAPDH) (TaqMan Gene Expression Assay, Applied Biosystems) acts as the endogenous control in this expression study.

### Statistical Analysis

IBM SPSS 20 was used to analyze the correlation of BACH1 expression to age, sex, red blood cell indices, HbF, HbA2 level, Xmn1 genotype, β-thalassemia genotypes, HO-1 expression, and α-, β-, and γ-globin genes expression. BACH1 was also correlated to clinical data such as transfusion history, splenomegaly, iron overloading, and iron chelation therapy. The parameters were analyzed using analysis of variance, Student’s t-test, Pearson’s correlation coefficient analysis, and simple linear regression analysis.

## RESULTS

### Correlation of BACH1 Gene Expression with Age, Sex, and Red Blood Cell Parameters

A total of 47 unrelated HbE/beta-thalassemia samples were collected (14 males and 33 females; 45 Malays and 2 Chinese; 21-57 years old). These individuals had thalassemia intermedia with a mean hemoglobin level of 7.40±2.56 g/dL. Co-inheritance of α-thalassemia or iron-deficiency anemia was not found in our sample cohort. BACH1 expression in our samples varied up to 100.43 with a mean of 1.601±42.775 fold change and was positively skewed. To fit the normal distribution curve for statistical analyses, the BACH1 expression results were log-transformed with approximately 2 log-fold variation. Simple linear regression analysis showed positive association of log BACH1 expression with age (p=0.006; R2=0.155). Log BACH1 expression was not correlated to sex or most red blood cells parameters, except reticulocyte count (p=0.005; R2=0.16) and percentage (%) (p=0.01; R2=0.138). Detailed results can be found in Table 1.

### Correlation of BACH1 Gene Expression to Hemoglobin +E/β-Thalassemia Genotypes, BACH1 Genotypes, and Xmn1 Polymorphisms

The β-thalassemia mutations present in these HbE/β-thalassemia individuals were 20 IVS I-5 (G>C), 18 IVS I-1 (G>T), 7 CD41/42 (-TCTT), and 2 IVS II-654. BACH1 expression was not correlated to any β-thalassemia genotypes (p=0.6531) or Xmn1 polymorphisms (p=0.139; R2=0.031) (Table 1). The Xmn1 -/- polymorphism was excluded from the analysis as the numbers were too small (n=3). The efficiency of βE-globin mRNA splicing could be the main factor of β-globin expression variations, rather than β genotypes, to affect the BACH1 expression level [12]. Five samples were randomly selected for re-sequencing of the BACH1 gene. However, no polymorphisms could be detected in these samples. Polymorphisms that affect the BACH1 gene expression could be located upstream in the enhancer regions that were not sequenced.

### Correlation of BACH1 Gene Expression with Globin Gene Expressions and Heme Oxygenase 1 Expression

**Figure 1:**
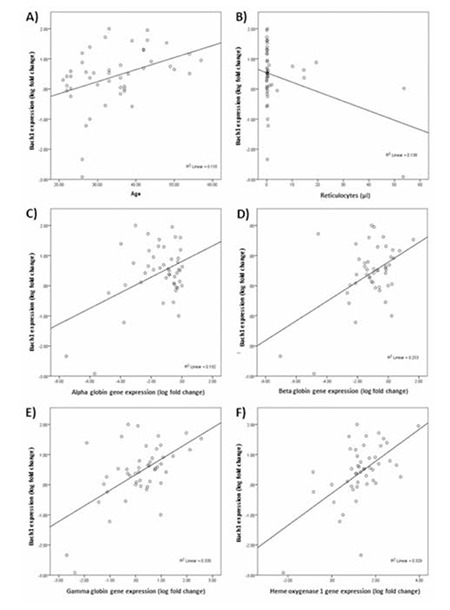
Correlations of BACH1 expression with age; reticulocyte count; α-, β-, and γ-globin gene expression; and heme oxygenase 1 gene expression. A) Correlation of BACH1 with age (p=0.006), B) correlation of BACH1 with reticulocyte number (µL) (p=0.01), C) correlation of BACH1 with α-globin expression (p=0.002), D) correlation of BACH1 with β-globin expression (p=0.001), E) correlation of BACH1 with γ-globin expression (p=0.001), F) correlation of BACH1 expression with heme oxygenase 1 expression (p=0.001).

All positively skewed expression data were log-transformed to fit the normal distribution. Log BACH1 expression was positively correlated to log α-globin (p=0.002; R2=0.192), log β-globin (p=0.001; R2=0.253), log γ-globin (p=0.001; R2=0.330), and log HO-1 (p=0.001; R2=0.329) gene expressions (Figure 1).

### Correlation of BACH1 Gene Expression to Clinical Data

**Figure 2:**
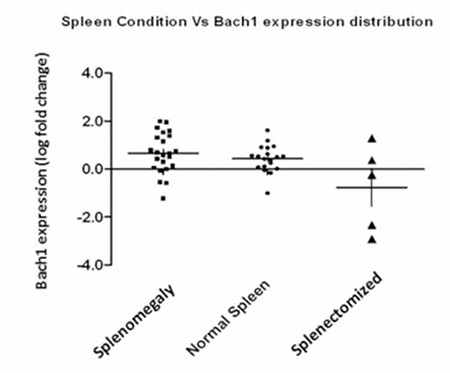
BACH1 expression correlation to spleen sizes and splenectomized hemoglobin E/β-thalassemia individuals (p=0.0085).

BACH1 was not affected by transfusion history (p=0.6298), iron overloading (p=0.6216), or iron chelation therapy (p=0.1743). However, BACH1 correlation was significant in splenectomized individuals (p=0.0085) (Figure 2).

## DISCUSSION

The heme-BACH1 transcription activation pathway provides a mechanism for the cellular environment to maintain a balanced homeostasis during erythroid differentiation when the production and assembly of hemoglobin actively comes together. While BACH1 functions to maintain a dormant expression of the α- and β-globin genes, it could quickly change with the increase of heme [13]. However, in HbE/β-thalassemia, the expression of the β-globin gene is reduced. We studied the BACH1 gene expression in this disorder to elucidate the function of BACH1 when the α/β-globin chain ratio balance is compromised.

HbE/β-thalassemia red cells are known to have increased levels of oxidative stress, which is caused by the excess free α-globin chains available and the increased labile iron pool [14,15]. Cell damage or death caused by increased available reactive oxygen species could be related to the natural cellular aging process or iron overload in HbE/β-thalassemia as excess iron absorption from the intestinal tract accumulates slowly over time [15,16,17,18]. A study by Dohi et al. showed that BACH1 is involved in the repression of premature cellular senescence induced by oxidative stress [19]. Therefore, the rise of BACH1 expression as age increases is perhaps to delay premature cellular aging due to excessive oxidative stress.

As reticulocyte numbers reflect the bone marrow erythropoietic activity, the degree of ineffective erythropoiesis due to harmful precipitation of excess free α-globin chains in β-thalassemia can be phenotypically typed [20,21,22]. The negative correlation of BACH1 and reticulocyte count could reflect the role of BACH1 in repressing the excess α-globin gene to reduce the rate of ineffective erythropoiesis [6].

HbE/β-thalassemia does not only have lower βE-globin chain synthesis but also the presence of aberrantly spliced βE-globin mRNA. Higher levels of aberrant compared to correctly spliced βE-globin mRNA have been linked to increased severity [12,23]. BACH1 could function to decrease the production of βE-globin to reduce the burden of aberrantly spliced βE-globin mRNA. The non-association of beta genotypes to BACH1 expression could be due to the masking of the genotype expression by HbE.

In terms of the lack of β-globin gene expression in HbE/β-thalassemia, compensatory increase of γ-globin chains to form HbF with excess α-globin chains explains the indirect correlation to BACH1 expression. To date, there has not been any study done to examine the effect of BACH1 on the γ-globin gene. However, if BACH1 does have a repressive role on γ-globin gene expression, then BACH1 is maintaining cellular homeostasis by regulating the expression of the γ-globin chain. Wickramasinghe and Lee showed that large production of γ-globin chains does not necessarily protect against extensive precipitations of α-globin monomers [24].

BACH1 is also a well-known repressor of HO-1. De Franceschi et al. showed that heme and HO-1 levels were both reduced in β-thalassemia precursors compared to controls [8]. BACH1 could be repressing HO-1 in β-thalassemia to prevent the cytotoxic effect of excess free heme due to the lack of normal hemoglobin formation and also to prevent the excessive accumulation of heme degradation products as they possess the potential to be cytotoxic beyond a certain threshold [25]. However, this mechanism could be overwhelmed in splenectomized patients. Splenectomy is performed when accelerated transfusions are required to maintain adequate hemoglobin levels in the patient [26]. A study on thalassemia intermedia patients showed severe iron decompartmentalization in the red blood cells of splenectomized patients compared to non-splenectomized patients with significantly higher levels of membrane-bound free iron, non-heme iron, and heme compounds [27]. Heme not only suppresses the function of BACH1 but has also been found to promote BACH1 degradation [28].

## CONCLUSION

BACH1 plays a role in maintaining the microcellular homeostasis in HbE/β-thalassemia by repressing the excess α-globin chains and aberrantly spliced βE-globin mRNA as well as preventing cytotoxic effects of excess free heme, although the amount expressed may not be sufficient to alleviate the severity of the HbE/β-thalassemia phenotype and it is abolished in the presence of excessive heme. Further investigations to confirm the pathways involved are necessary, perhaps by using a mouse model.

## Figures and Tables

**Table 1:**
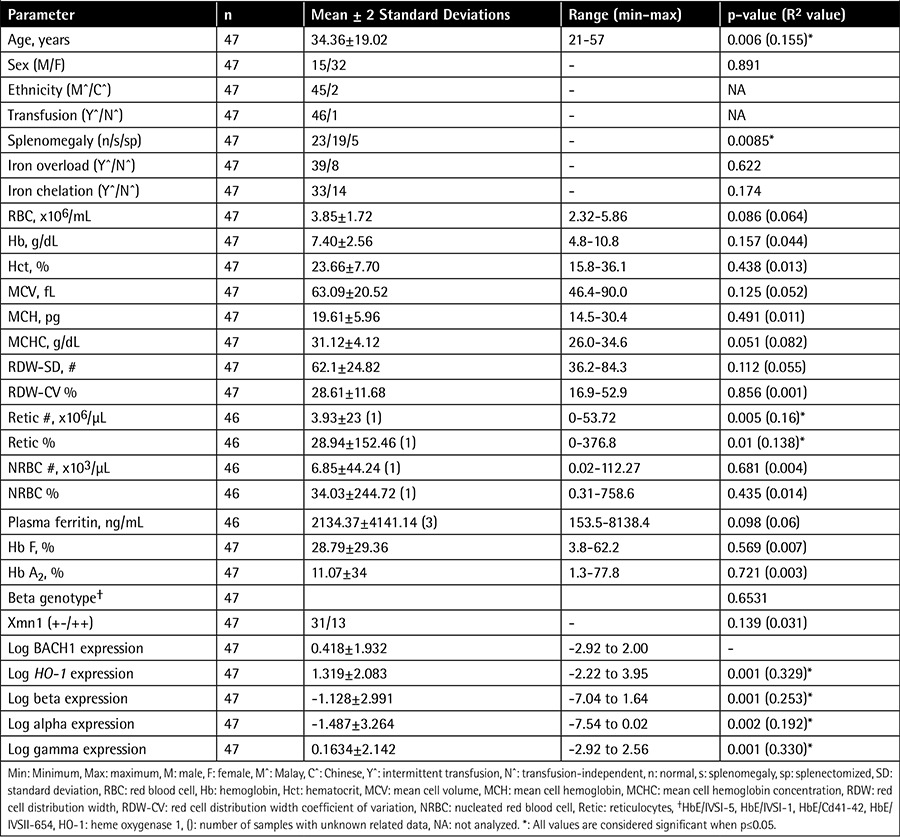
Correlation of BACH1 expression with demographic and hematological data and heme oxygenase 1 and globin gene expressions.
